# Suppression of heparan sulfation re-sensitizes YAP1-driven melanoma to MAPK pathway inhibitors

**DOI:** 10.1038/s41388-022-02400-z

**Published:** 2022-07-07

**Authors:** Sebastian M. Dieter, Domenica Lovecchio, Abhijeet Pataskar, Martina K. Zowada, Pierre-René Körner, Anna Khalizieva, Olaf van Tellingen, Dirk Jäger, Hanno Glimm, Reuven Agami

**Affiliations:** 1 Division of Oncogenomics, Oncode Institute, The Netherlands Cancer Institute, Amsterdam, The Netherlands; 2 Translational Functional Cancer Genomics, National Center for Tumor Diseases (NCT) Heidelberg and German Cancer Research Center (DKFZ), Heidelberg, Germany; 3 Department of Medical Oncology, Department of Medical Oncology, National Center for Tumor Diseases (NCT), Heidelberg University Hospital, Heidelberg, Germany; 4 Department of Translational Medical Oncology, National Center for Tumor Diseases (NCT) Dresden and German Cancer Research Center (DKFZ), Dresden, Germany; 5 Division of Pharmacology, The Netherlands Cancer Institute, Amsterdam, The Netherlands; 6 German Cancer Consortium (DKTK), Heidelberg, Germany; 7 Center for Personalized Oncology, National Center for Tumor Diseases (NCT) Dresden and University Hospital Carl Gustav Carus Dresden at TU Dresden, Dresden, Germany; 8 German Cancer Consortium (DKTK), Dresden, Germany; 9 Erasmus MC, Rotterdam University, Rotterdam, The Netherlands

**Keywords:** Cancer genetics, Skin cancer

## Abstract

Accumulating evidence identifies non-genetic mechanisms substantially contributing to drug resistance in cancer patients. Preclinical and clinical data implicate the transcriptional co-activators YAP1 and its paralog TAZ in resistance to multiple targeted therapies, highlighting the strong need for therapeutic strategies overcoming YAP1/TAZ-mediated resistance across tumor entities. Here, we show particularly high YAP1/TAZ activity in MITF^low^/AXL^high^ melanomas characterized by resistance to MAPK pathway inhibition and broad receptor tyrosine kinase activity. To uncover genetic dependencies of melanoma cells with high YAP1/TAZ activity, we used a genome-wide CRISPR/Cas9 functional screen and identified SLC35B2, the 3′-phosphoadenosine-5′-phosphosulfate transporter of the Golgi apparatus, as an essential gene for YAP1/TAZ-driven drug resistance. SLC35B2 expression correlates with tumor progression, and its loss decreases heparan sulfate expression, reduces receptor tyrosine kinase activity, and sensitizes resistant melanoma cells to BRAF inhibition in vitro and in vivo. Thus, targeting heparan sulfation via SLC35B2 represents a novel approach for breaking receptor tyrosine kinase-mediated resistance to MAPK pathway inhibitors.

## Introduction

Targeted drugs have substantially improved the outcome of patients with advanced malignancies [1–3]. However, resistance inevitably occurs and drastically limits patient survival. Genetic and non-genetic mechanisms contribute to drug resistance in cancer [4], and understanding the underlying molecular principles is of pivotal importance to achieve meaningful improvements in treatment responses.

Much research has focused on genetic alterations as mediators of drug resistance, but recent evidence highlights the substantial role of transcriptional, non-genetic adaptation of cancer cells on drug treatment as a driver of resistance [5–7]. YAP1 and its paralog TAZ, the transcriptional co-activators and downstream effectors of the Hippo pathway, share transcriptional programs driving malignant transformation, tumor progression [8], and a drug-tolerant cancer cell state, associated with resistance toward multiple targeted drugs, chemo-, and radiotherapies [9–11].

As genetic alterations within the Hippo pathway including YAP1 and TAZ are rather rare across tumor types and do not comprehensively identify YAP1/TAZ-driven tumors, target gene signatures have been inferred from genetic interference experiments, transcriptomic and epigenomic analysis [12**–**13]. These signatures allow for quantitative assessment of YAP1/TAZ activity and have, in part, been applied to identify tumor entities that display high YAP1/TAZ activity in larger cancer patient cohorts. However, YAP1/TAZ-driven patient tumors are poorly defined phenotypically and functionally so far, and vulnerabilities associated with high YAP1/TAZ activity in specific treatment contexts remain unclear.

YAP1/TAZ bind TEAD and additional transcription factors to induce transcription of target genes [14]. The interaction of YAP1/TAZ with TEAD transcription factors can be disrupted pharmacologically [15, 16]. However, the critical physiological functions of YAP1/TAZ in organ growth, stem cell amplification, and cell fate decisions as well as its integration in, e.g., the Wnt signaling pathway [17], might limit the suitability of YAP1/TAZ as a direct therapeutic target. Thus, understanding vulnerabilities associated with YAP1/TAZ activity will be crucial for therapeutic approaches overcoming YAP1/TAZ-associated drug resistance.

Here, we used BRAF^V600E^ mutant melanoma models with defined YAP1 activity to identify a clinically relevant subgroup of MAPK pathway inhibitor (MAPKi)-resistant melanoma characterized by high YAP1 activity. We further used a genome-wide CRISPR/Cas9 screen to identify vulnerabilities of YAP1/TAZ-driven melanoma cell lines and validated a sulfate transporter as an attractive therapeutic target whose inhibition overcomes YAP1/TAZ-driven mechanisms of MAPKi resistance.

## Results

### YAP1 induces resistance to BRAF and MEK inhibitors in BRAF^V600E^ mutant melanoma cell lines

**Fig. 1:**
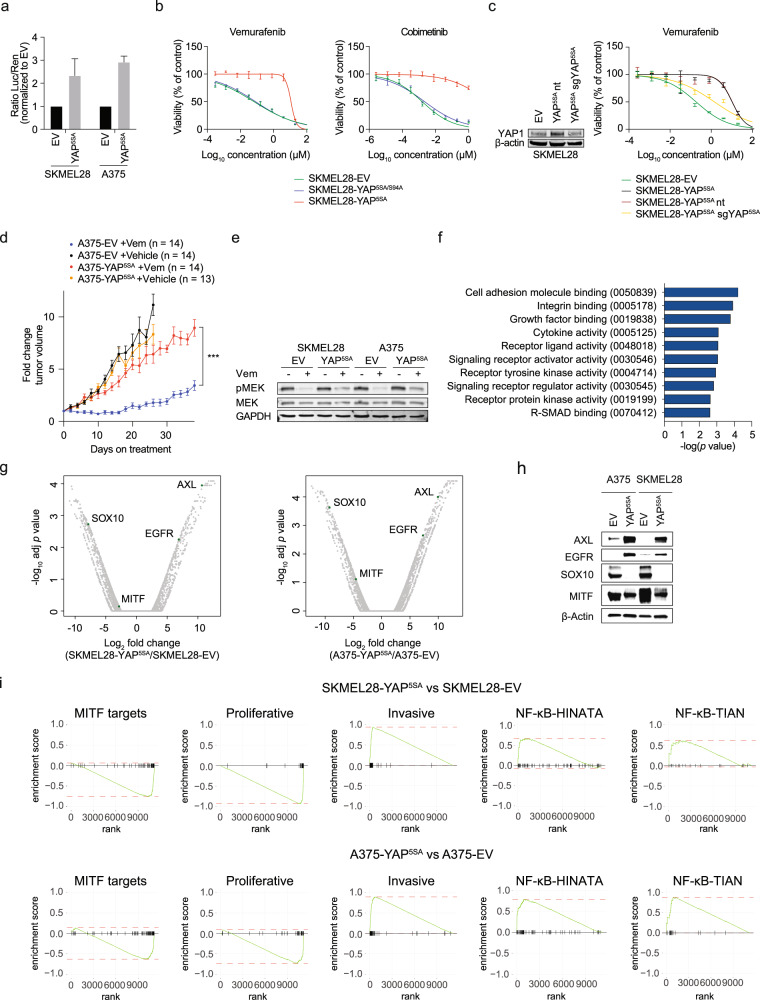
YAP1 confers MAPK pathway inhibitor resistance and induces the MITF^low^/AXL^high^ phenotype. **a** A TEAD luciferase reporter was used to quantify the transcriptional activity of YAP1 in engineered melanoma cell lines with constitutive YAP1 activity (YAP^5SA^) and empty vector (EV) controls (mean + SD of three replicates is presented). **b** Cell viability assays were used to determine the sensitivity of cell lines lentivirally infected with YAP^5SA^, transcriptionally inactive YAP^5SA/S94A^, or EV controls toward a BRAF inhibitor (Vemurafenib) and a MEK inhibitor (Cobimetinib; mean ± SD of three replicates is presented). **c** SKMEL28-YAP^5SA^ cell lines were lentivirally infected with an sgRNA targeting YAP^5SA^ or a non-targeting (nt) control. Immunoblotting with indicated antibodies was used to quantify YAP1 protein levels and sensitivity toward Vemurafenib was assessed (mean ± SD of three replicates is presented). **d** A375-EV and A375-YAP^5SA^ cells were injected into NSG mice and established tumors were treated with Vemurafenib (Vem; 50 mg kg^–1^ per day orally) or vehicle (mean ± SEM of fold change tumor volume compared to start of treatment is presented; *p* value from mixed-effect model. ***p < 0.001). **e** Analysis of MEK inhibition after treatment with 5 µM Vemurafenib for 24 h using immunoblotting with indicated antibodies. **f** GO term enrichment in genes significantly upregulated (*p* < 0.01) in A375-YAP^5SA^ and SKMEL28-YAP^5SA^ compared to respective EV controls as identified by RNA-seq. **g** Changes in expression of individual genes from the same experiment as **f**. **h** Immunoblotting of indicated antibodies for selected genes upregulated or suppressed upon YAP1 activation using protein lysates from A375-YAP^5SA^ and SKMEL28-YAP^5SA^ cell lines and corresponding EV controls. **i** Enrichment and depletion of gene sets for MITF targets, genes of the proliferative and invasive state, and NF-κB targets in SKMEL28-YAP^5SA^ and A375-YAP^5SA^ compared to EV controls. **a**–**c**, **e**, and **h** Experiments were performed at least two times independently, one representative experiment is shown. Western blot in **c** was performed once. **f**, **g**, **i** RNA was isolated from two biological replicates per cell line.

We transduced the BRAF^V600E^ mutant melanoma cell lines A375 and SKMEL28 with a lentiviral vector expressing a constitutively active mutant of YAP1 (YAP^5SA^) to induce YAP1 activity, which was confirmed using a luciferase reporter (Fig. 1a) [18]. In line with previous reports, cell viability assays showed that YAP^5SA^ expression confers resistance to Vemurafenib and Cobimetinib, a BRAF^V600E^ and a MEK inhibitor, respectively (Fig. 1b) [19]. This effect was mediated by TEAD transcription factors, as the addition of S94A mutation to YAP^5SA^ (YAP^5SA/S94A^), which is known to disrupt the binding of YAP1 to TEAD [20], abolished YAP^5SA^-induced drug resistance (Fig. 1b). As expected, YAP^5SA^-mediated resistance was reversible by knockout of YAP^5SA^ (Fig. 1c), even though reversibility was incomplete, most likely due to limited knockout efficiency. Furthermore, tumors formed by YAP^5SA^-activated melanoma cells in immunodeficient NSG mice grew despite Vemurafenib treatment, confirming YAP1-induced resistance to Vemurafenib in vivo (Fig. 1d).

YAP1/TAZ induce the expression of multidrug transporters, thereby potentially increasing drug efflux [21]. To exclude increased drug efflux underlying YAP^5SA^-mediated MAPKi resistance, we assessed MEK phosphorylation in YAP^5SA^ melanoma cell lines, as efficient BRAF^V600E^ inhibition was expected to reduce MEK phosphorylation. Indeed, Vemurafenib reduced MEK phosphorylation similarly in both control and YAP^5SA^ cell lines (Fig. 1e), excluding increased drug efflux as a likely mechanism for YAP^5SA^-induced drug resistance.

### YAP1 activates transcription of genes related to growth factor signaling

RNA-seq identified genes significantly upregulated in YAP^5SA^ cells compared to controls (adj *p* < 0.01) and Gene Set Enrichment Analysis (GSEA) revealed enrichment of multiple gene ontology (GO) terms related to cell surface receptor signaling, including growth factor binding and activity regulation (Fig. 1f and Supplementary Table 1). Top differentially expressed genes included the receptor tyrosine kinases (RTKs) AXL and EGFR, both known to contribute to MAPKi resistance (Fig. 1g) [22**–**23] and overexpressed at the protein level as well (Fig. 1h).

Several reports identified an inverse correlation between transcript levels of AXL and the transcription factor MITF in a melanoma subset characterized by marked resistance to MAPKis [23, 24]. In addition, The Cancer Genome Atlas (TCGA) analysis has identified three transcriptomic subclasses of melanomas with distinct survival rates, one of them being defined by low MITF expression [25]. Moreover, melanoma cell lines switch between an MITF-driven proliferative and a YAP1-driven invasive state which both have been demonstrated experimentally to be associated with respective gene signatures [26, 27]. Interestingly, MITF and its targets were downregulated in YAP^5SA^ cells (Fig. 1i). Moreover, YAP^5SA^ melanoma cells showed a sharp depletion of the proliferative and enrichment of the invasive gene signature, highlighting YAP1/TAZ as a potential driver of these phenotype switches (Fig. 1i).

MITF^low^/AXL^high^ melanoma cell lines and tumors show expression patterns of active NF-κB signaling [24]. We observed an enrichment of NF-κB targets in YAP^5SA^ cells compared to controls (Fig. 1i), suggesting YAP1 as a transcriptional activator of NF-κB signaling in melanoma cells.

### YAP1 is highly active in MITF^low^/AXL^high^ melanoma cell lines and patient tumors

In a previous study, expression of MITF and its target genes were best correlated with sensitivity to BRAF inhibitors in vitro, whereas AXL, TPM1, NRP1, and CDH13 correlated well with resistance [24]. While sensitivity marker genes were suppressed in YAP^5SA^ melanoma cells, resistance markers were upregulated (Supplementary Fig. 1), supporting the role of YAP1 as a molecular driver of the MITF^low^/AXL^high^-resistance phenotype in melanoma.

**Fig. 2:**
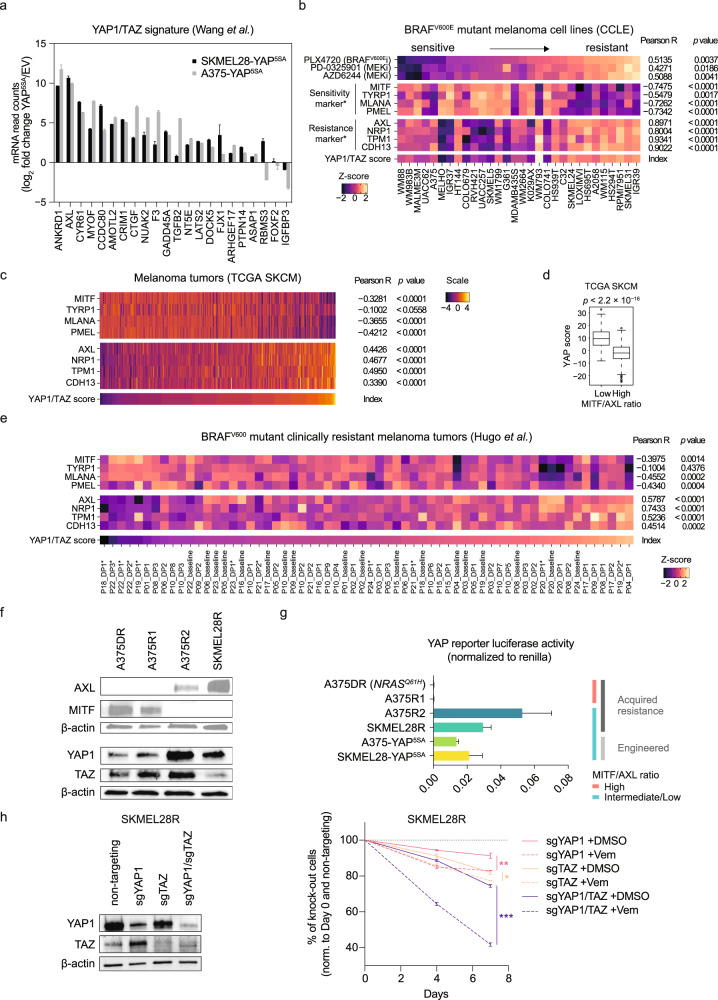
YAP1 activity is associated with the MITF^low^/AXL^high^ melanoma cell state and MAPKi resistance. **a** Fold change of mRNA levels of the 22 genes from the YAP1 signature [28] in YAP^5SA^ melanoma cells compared to controls (mean + SD of two biological replicates per cell line is presented). **b** Correlation of drug sensitivity of BRAF^V600E^ mutant melanoma cell lines (*n* = 30) and expression of known sensitivity and resistance marker genes (marked by asterisks) [24] as well as the YAP1/TAZ score (*p* values from unpaired, two-tailed *t*-tests; BRAF^V600E^i, BRAF^V600E^ inhibitor; MEKi, MEK inhibitor). **c** Correlation of sensitivity and resistance genes and the YAP1/TAZ score across the TCGA melanoma cohort (SKCM; *p* values from unpaired, two-tailed *t*-tests). **d** Comparison of YAP1/TAZ score of tumors of the TCGA melanoma cohort with low versus high MITF/AXL ratio (*n* = 236, respectively; *p* value from unpaired, two-tailed *t*-test). **e** Correlation of sensitivity and resistance genes and the YAP1 score across a melanoma patient cohort [30] (*n* = 19) treated with BRAF and/or MEK inhibitors (asterisks indicate patient tumors treated with a combination of a BRAF and MEK inhibitor; DP, disease progressive). **f** Immunoblotting of indicated antibodies using protein lysates from melanoma cell lines with acquired MAPKi resistance. **g** YAP1 activity was determined in melanoma cell lines with acquired MAPKi resistance and engineered YAP^5SA^ cell lines using a luciferase reporter assay (mean + SD of at least six replicates are presented). **h** YAP1, TAZ, and simultaneous YAP1/TAZ CRISPR/Cas9-mediated knockout cell lines were generated and protein levels determined using immunoblotting with indicated antibodies (left). Cell lines were tested for sensitivity toward 2 µM vemurafenib compared to DMSO in eGFP competition assays mixed with SKMEL28R non-targeting controls, respectively (right). The eGFP ratio was determined at different time points as indicated using flow cytometry (mean ± SD of two replicates is presented; *p < 0.05, **p < 0.01, ***p < 0.001. **f**–**h** These experiments have been performed at least two times independently, one representative experiment is shown; competition assay in **h** was performed once with two replicates).

To corroborate the role of YAP1/TAZ as an upstream regulator of the MITF^low^/AXL^high^ melanoma subclass, and as a driver of resistance to MAPKis across melanoma cell lines and patient tumors, we scored for YAP1/TAZ activity using an established pancancer YAP1/TAZ target gene signature (Fig. 2a and Supplementary Table 2) [28]. Almost all 22 YAP1/TAZ target genes of this signature were overexpressed in YAP^5SA^ cells, and similar results were observed for additional independent YAP1/TAZ target gene signatures (Fig. 2a and Supplementary Fig. 2a), confirming functional YAP1 activation in YAP^5SA^ cells [28]. Across 30 BRAF^V600E^ mutant melanoma cell lines that were screened against BRAF and MEK inhibitors within the Cancer Cell Line Encyclopedia (CCLE) project [29], the YAP1/TAZ 22 target gene score correlated significantly with resistance to MAPK inhibitors, the MITF^low^/AXL^high^ phenotype, and the expression of known resistance marker genes (Fig. 2b) [24]. In addition, the correlation of YAP1/TAZ with the MITF^low^/AXL^high^ phenotype was significant across the TCGA melanoma tumors and clinically resistant melanoma samples amongst which a subset of resistant tumors acquired YAP1 activity along with clinical resistance toward MAPK inhibitors (Fig. 2c–e) [30]. Interestingly, some CCLE melanoma cell lines showed intermediate YAP1/TAZ scores, but high MAPKi sensitivity (Fig. 2b). In these cell lines, the expression of MITF and its target genes was maintained, indicating that YAP1/TAZ-associated MITF suppression is context-dependent and required for MAPKi resistance.

To confirm high YAP1 activity in MAPKi-resistant MITF^low^/AXL^high^ melanoma cells independent from YAP^5SA^ engineered YAP1 activation, we used a YAP1/TAZ-reporter luciferase assay in melanoma cell lines with spontaneously acquired resistance to a BRAF^V600E^ inhibitor (SKMEL28R, A375R1, A375R2) or the combination of a BRAF^V600E^ and a MEK inhibitor (A375DR) [23, 31]. Indeed, two MITF^low^/AXL^intermediate/high^ resistant cell lines demonstrated higher YAP1/TAZ activity compared to resistant MITF^high^/AXL^low^ cell lines (Fig. 2f, g). In addition, YAP1 levels were increased in MITF^low^/AXL^intermediate/high^ cell lines (Fig. 2f), whereas TAZ was either increased or decreased, suggesting a particular role for YAP1 in these cell lines. Treatment of A375R2 and SKMEL28R cells with the YAP1/TAZ inhibitor verteporfin suppressed expression of resistance markers (Supplementary Fig. 2b), providing evidence for these genes being direct YAP1/TAZ targets. MITF was either unaffected or slightly suppressed upon Verteporfin treatment, suggesting its suppression by YAP1/TAZ being an indirect effect.

Interestingly, the MITF^low^/AXL^intermediate/high^ melanoma cell lines showed higher YAP1/TAZ activity compared to the engineered YAP^5SA^ cell lines, indicating that the latter reflect a physiological level of high YAP1 activity (Fig. 2g).

To probe the dependency of MAPKi-resistant MITF^low^/AXL^high^ melanoma cell lines on YAP1 and TAZ, we generated cell lines with knockout of either YAP1, TAZ, or both (Fig. 2h). Single gene knock-outs showed modest re-sensitization to Vemurafenib, whereas simultaneous knockout of both YAP1 and TAZ revealed pronounced re-sensitization in MITF^low^/AXL^high^ melanoma cells (Fig. 2h). Thus, even though YAP1 levels are increased in MITF^low^/AXL^high^ cells, TAZ might compensate at least in part for YAP1 loss.

Altogether, our data indicate that YAP1/TAZ induce the MAPKi-resistant MITF^low^/AXL^high^ phenotype in melanoma cells and likely contribute as upstream regulators to MAPKi resistance of BRAF^V600E^ melanoma tumors in the clinic. While a role of YAP1/TAZ for MAPKi resistance has been previously described [19], we extend this knowledge by linking high YAP1/TAZ activity with the distinct MITF^low^/AXL^high^ melanoma subset across large numbers of melanoma cell lines and patient tumors and identify this phenotype as a potential biomarker predicting response to therapeutic approaches exploiting YAP1/TAZ-associated vulnerabilities in melanoma.

### A genome-wide CRISPR/Cas9 screen identifies vulnerabilities of melanoma cells with high YAP1 activity

**Fig. 3:**
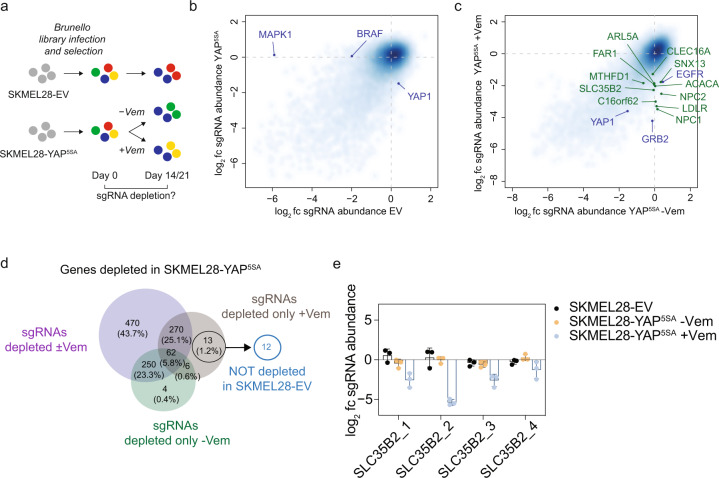
A genome-wide CRISPR/Cas9 screen identifies genetic dependencies of melanoma cells with high YAP1 activity. **a** Experimental layout of genome-wide screen for genetic dependencies of SKMEL28-YAP^5SA^ melanoma cells ± Vemurafenib (Vem) treatment and EV control cells (the experiment was performed with three replicates per cell line). **b** Change in sgRNA abundance (end of the experiment compared to start) per gene comparing SKMEL28-YAP^5SA^ cells without treatment and SKMEL28-EV cells (blue labels indicate expected hits; fc, fold change). **c** Change in sgRNA abundance per gene comparing SKMEL28-YAP^5SA^ cells treated with or without 5 µM Vemurafenib (blue labels indicate expected hits; green labels indicate hits selected for validation). **d** Strategy for selection of hits for validation focusing on genes whose sgRNAs were all only essential for Vemurafenib-treated SKMEL28-YAP^5SA^ cells. **e** Change in sgRNA abundance for SLC35B2 in screened SKMEL28 cell lines (mean ± SD of three replicates is presented).

As the engineered YAP1-activated melanoma cell lines modeled a clinically relevant mode of drug resistance, we sought to systematically identify vulnerabilities in these cells using a genome-wide CRISPR/Cas9 screen. SKMEL28-YAP^5SA^ and SKMEL28 empty vector (EV) control cells were transduced with the Brunello CRISPR/Cas9 library, which comprises approximately 80,000 unique sgRNAs targeting nearly 20,000 genes with multiple sgRNAs per gene, and were selected with Puromycin (Fig. 3a) [32]. Two arms included SKMEL28-YAP^5SA^ cells in the presence or absence of Vemurafenib treatment, respectively, and one arm included untreated SKMEL28-EV cells. After selection, cells were propagated for 2–3 weeks, ensuring the same number of cell divisions per cell line, and harvested, genomic DNA was isolated, and the abundance of each sgRNA at the end of the experiment was compared to the start.

Differential abundance analysis in control SKMEL28-EV cells revealed significant depletion of BRAF and MAPK1 (also known as ERK2) targeting sgRNAs, in line with the known addiction of BRAF^V600E^ mutant melanomas to the MAPK pathway (Fig. 3b and Supplementary Table 3). In contrast, sgRNAs targeting these genes were not depleted in SKMEL28-YAP^5SA^ cells, confirming the loss of addiction to the oncogenic MAPK pathway. Amongst YAP1/TAZ target genes overexpressed in both YAP^5SA^ melanoma cell lines, only VGLL3 scored as essential in untreated YAP^5SA^ melanoma cells.

We further compared sgRNA distribution in SKMEL28-YAP^5SA^ cells in the presence or absence of Vemurafenib treatment, which revealed the depletion of several genes only in Vemurafenib-treated cells, including the YAP1/TAZ target gene EGFR, and GRB2 (Fig. 3c). EGFR expression has been shown to mediate melanoma resistance toward BRAF^V600E^ inhibitors [22] and not to be beneficial in the absence of Vemurafenib. GRB2 is an adaptor protein linking activated cell surface receptors including EGFR to downstream targets and is expected to re-sensitize SKMEL28-YAP^5SA^ cells to Vemurafenib.

Lastly, when we searched for sgRNAs depleted only in SKMEL28-YAP^5SA^, but not in control SKMEL28-EV cells, a large number of sgRNAs targeting genes involved in RNA processing, transcription as well as mRNA translation, and hence genes generally essential for cell proliferation, was uncovered (Supplementary Fig. 3a). Thus, our screen identified expected hits, which indicates the technical reliability of the screen, and permitted the identification of essential genes in YAP1-activated cells. Notably, genes known to be implicated in YAP1 activation, e.g., integrins or SRC [33, 34], did not score in our screen, as we were using the constitutive active YAP^5SA^ mutant to simulate YAP1, which precluded the identification of genes essential for MAPKi-resistance upstream of YAP1/TAZ.

Next, we focused on 12 genes of which all targeting sgRNAs were significantly depleted in SKMEL28-YAP^5SA^ cells after treatment, but not in untreated SKMEL28-YAP^5SA^ or control SKMEL28-EV cells (Fig. 3d). Interestingly, six of these genes were related to lipid metabolism (Supplementary Fig. 3b), which has been implicated in drug resistance before [35]. We validated the 12 hits in SKMEL28-YAP^5SA^ cells and A375-YAP^5SA^ cells using growth competition assays. While many hits could be confirmed in SKMEL28-YAP^5SA^ cells, only EGFR and SLC35B2 were validated in A375-YAP^5SA^ cells (Fig. 3e and Supplementary Fig. 3c). sgRNAs targeting EGFR or SLC35B2, respectively, were depleted only in YAP^5SA^ cells treated with Vemurafenib, but were not substantially changed in untreated YAP^5SA^ cells or parental melanoma cells (EV). In contrast to EGFR, SLC35B2 has not been investigated in the context of cancer and drug resistance. We thus further characterized SLC35B2.

### SLC35B2 is required for YAP1-mediated melanoma cell resistance to Vemurafenib in vitro and in vivo

**Fig. 4:**
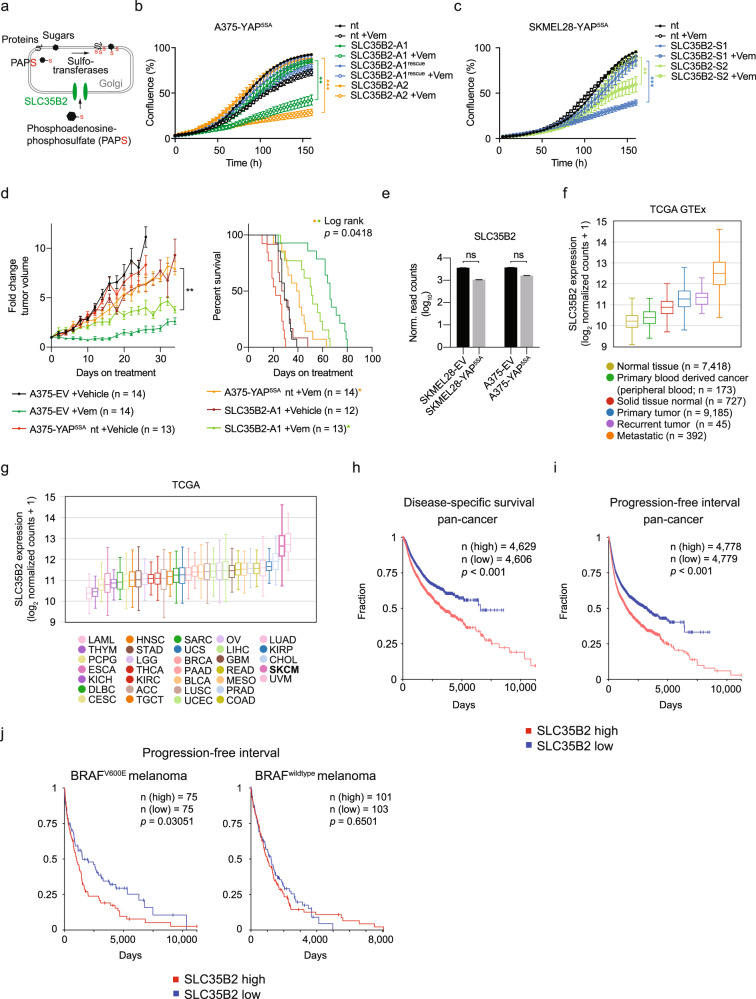
YAP1-mediated MAPKi resistance depends on SLC35B2. **a** Function of SLC35B2 as a PAPS transporter in the Golgi apparatus. **b**, **c** Confluency of A375-YAP^5SA^ (**b**) and SKMEL28-YAP^5SA^ (**c**) cell clones with knockout of SLC35B2 ± treatment with 5 µM Vemurafenib (Vem) compared to nt control cells and cells with expression of a codon-optimized SLC35B2 which was resistant to the SLC35B2-targeting sgRNA (rescue; mean ± SD of at least three replicates is presented; *p* values from unpaired, two-tailed *t*-tests; **p < 0.005, ***p < 0.001; experiments were performed with at least three replicates two times independently, one representative experiment is shown). **d** A375-YAP^5SA^ cells with SLC35B2 knockout and controls were injected in NSG mice and treated with Vemurafenib (50 mg kg^–1^ per day orally) or vehicle upon tumor formation (mean ± SEM are presented; *p* values from mixed-effect model; **p < 0.01). **e** mRNA levels of SLC35B2 in YAP^5SA^ melanoma cells compared to EV controls (mean ± SD of two biological replicates is presented; *p* values from unpaired, two-tailed *t*-tests; Norm., normalized; ns, not significant). **f** Box plot presenting expression of SLC35B2 across normal tissues (GTEx datasets), tumor-associated normal tissue (solid tissue normal) as well as primary, recurrent and metastatic tumors (TCGA datasets) as assessed by RNA-seq. **g** Box plot presenting SLC35B2 expression levels across tumor entities (TCGA; *n* = 33). Data for SKCM are highlighted in bold. **h**, **i** Association of disease-specific survival (**h**) and PFI (**i**) with SLC35B2 expression level in the TCGA pancancer cohort (*p* values from log-rank test). **j** Association of PFI with SLC35B2 expression level in metastatic melanomas (TCGA SKCM; *p* values from log-rank test).

SLC35B2 is a transporter of the activated sulfate donor, 3’-phosphoadenosine-5’-phosphosulfate (PAPS), and is localized in the membrane of the Golgi apparatus (Fig. 4a) [36]. SLC35B2 carries PAPS from the cytosol into the lumen of the Golgi apparatus, where it is used in sulfation reactions for the modification of proteins and proteoglycans [37]. It has been described as an attractive therapeutic target to prevent viral infections, as SLC35B2 knockout did not affect the viability of normal cells, but prevented viral infection due to decreased sulfation of proteins or proteoglycans essential for viral entry [38–40].

To confirm that Vemurafenib-treated YAP^5SA^ melanoma cells rely on SLC35B2, we generated two monoclonal cell populations with CRISPR/Cas9-mediated knockout of SLC35B2 in SKMEL28-YAP^5SA^ (abbreviated as SLC35B2-S1 and SLC35B2-S2) and A375-YAP^5SA^ (abbreviated as SLC35B2-A1 and SLC35B2-A2) cells, respectively. Efficient knockout was confirmed by sequencing of the CRISPR/Cas9 target site (Supplementary Fig. 4), as validated antibodies against SLC35B2 are lacking. We then compared proliferation of these SLC35B2 knockout clones with YAP^5SA^ melanoma cells transduced with a non-targeting (nt) sgRNA. Vehicle-treated SLC35B2 knockout clones proliferated similar to nt controls (Fig. 4b, c). In contrast, upon treatment with Vemurafenib, SLC35B2 knockout clones of both cell types proliferated significantly slower, indicating that loss of SLC35B2 re-sensitized YAP^5SA^ melanoma cells to Vemurafenib in vitro. This phenotype could be reversed by ectopic expression of codon-optimized SLC35B2 which was resistant to the SLC35B2 targeting sgRNA (Fig. 4b), indicating the importance of SLC35B2 in YAP1-mediated resistance.

To recapitulate these findings in vivo, we transplanted SLC35B2 knockout and control cell populations subcutaneously in NSG mice. Following tumor formation, mice were treated with Vemurafenib. Similar to the observations in vitro, loss of SLC35B2 significantly decreased tumor growth and improved survival compared with controls upon treatment with Vemurafenib (Fig. 4d). Thus, loss of SLC35B2 sensitizes YAP^5SA^ melanoma cells to Vemurafenib in vitro and in vivo.

### SLC35B2 expression correlates with tumor progression and poor survival

As SLC35B2 has not been studied systematically in the context of cancer, we further assessed SLC35B2 expression in SKMEL28-YAP^5SA^ and A375-YAP^5SA^ cell lines and across human tumors using pancancer and normal tissue transcriptome data from TCGA and Genotype-Tissue Expression (GTEx) datasets [41]. We found a trend toward lower SLC35B2 expression in YAP^5SA^ cell lines compared to controls, indicating that SLC35B2 might not be a direct YAP1 target and molecular regulators independent from YAP1/TAZ might drive SLC35B2 expression in cancer (Fig. 4e). Interestingly, SLC35B2 was higher expressed in solid tumors compared to normal tissue, and its expression increased from primary to recurrent and to metastatic solid tumors, correlating with tumor progression (Fig. 4f). Furthermore, SLC35B2 was highly expressed in solid tumors, with the highest expression in cutaneous and uveal melanoma (Fig. 4g), and low expression in hematological malignancies. Importantly, high SLC35B2 expression across tumor entities was associated with significantly worse overall survival, disease-specific survival, and shorter progression-free interval (PFI), further highlighting its potential clinical relevance (Fig. 4h, i). Interestingly, high SLC35B2 expression in melanoma was significantly associated with shorter PFI in BRAF^V600E^ mutant, but not BRAF wildtype metastatic melanoma (Fig. 4j).

### SLC35B2 is required for efficient expression of heparan sulfate on the cell surface

SLC35B2 transports PAPS into the Golgi apparatus to serve as a substrate for sulfation of proteins and proteoglycans. The sulfated proteome is incompletely defined, but nearly all proteins experimentally shown to be sulfated were not expressed in A375 or SKMEL28 cells [42]. We thus focused on the impact of SLC35B2 on proteoglycans.

**Fig. 5:**
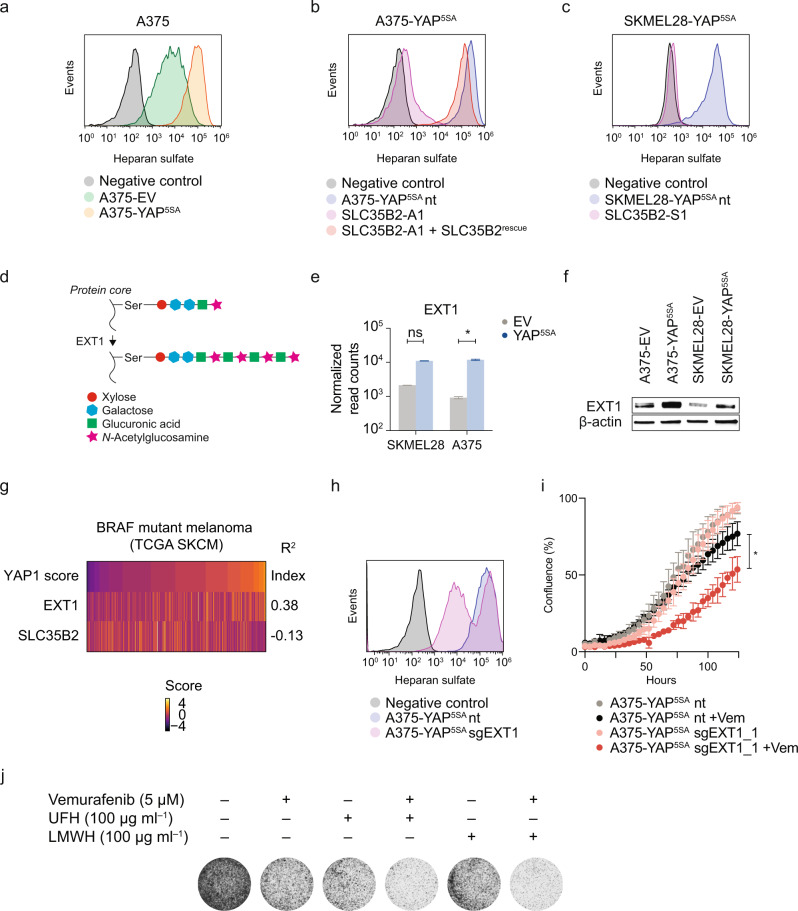
YAP1-mediated MAPKi resistance relies on SLC35B2-dependent HS expression. **a**–**c** HS expression at the cell surface as assessed by flow cytometry in A375-YAP^5SA^ and EV control cells (**a**) as well as A375-YAP^5SA^ (**b**) and SKMEL28-YAP^5SA^ (**c**) cell lines and SLC35B2 knockout clones (representative histograms of two independent experiments are shown; negative control, no primary antibody). **d** Role of EXT1 for HS synthesis. **e** EXT1 mRNA levels in YAP^5SA^ melanoma cells compared to EV controls (*p < 0.05*;* mean ± SD of two biological replicates are presented; *p* values from unpaired two-tailed *t*-tests). **f** EXT1 protein levels as assessed by immunoblotting in YAP^5SA^ melanoma cells compared to EV controls. **g** Correlation of YAP1/TAZ score with EXT1 and SLC35B2 expression across the TCGA cohort of BRAF mutant melanoma (*R*, Pearson’s correlation coefficient). **h** HS expression at the cell surface after EXT1 knockout compared to controls as assessed by flow cytometry (negative control, no primary antibody). **i** Confluency of A375-YAP^5SA^ cells transduced with sgRNAs targeting EXT1 or nt controls ± treatment with 5 µM Vemurafenib (Vem; mean ± SD of at least three replicates is presented; *p* values from unpaired, two-tailed *t*-tests; *p < 0.05). **j** Crystal violet staining after treatment of SKMEL28-YAP^5SA^ cells with Vemurafenib, unfractionated (UFH) or low molecular weight heparin (LMWH) or a combination thereof. All experiments were performed at least two times independently, one representative experiment is shown.

YAP^5SA^ melanoma cells showed transcriptional upregulation of genes related to growth factor and cytokine receptor signaling (Fig. 1f). Heparan sulfate (HS) is one of the most abundant proteoglycans and enhances growth factor signaling activity by stabilization of the complexes between growth factor receptors and their ligands [43]. Interestingly, using flow cytometry we observed that HS was strongly expressed on YAP^5SA^ cells, and A375-YAP^5SA^ cells revealed even stronger HS expression compared to EV controls (Fig. 5a). SKMEL28-EV cells, however, already displayed high HS levels similar to SKMEL28-YAP^5SA^ cells (Supplementary Fig. 5a). Notably, loss of SLC35B2 almost completely abrogated HS surface expression (Fig. 5b, c), which indicates that SLC35B2-dependent sulfation is critical for intact HS expression at the cell surface.

### Cell surface HS expression is required for efficient YAP1-mediated resistance to Vemurafenib

As SLC35B2 knockout might also affect sulfation of other proteins and proteoglycans, we sought to probe whether HS specifically contributes to Vemurafenib resistance. We examined other enzymes involved in HS biosynthesis and found EXT1, an endoplasmic reticulum protein-encoding glycosyltransferase involved in HS biosynthesis, to be overexpressed both at transcript and protein level in SKMEL28- and A375-YAP^5SA^ cells compared to controls (Fig. 5d–f), even though transcriptional upregulation was only statistically significant in A375-YAP^5SA^ cells. In addition, expression of EXT1, but not SLC35B2, was positively correlated with the YAP1/TAZ score in the TCGA cohort of BRAF mutant melanoma (Fig. 5g), suggesting that EXT1 is a YAP1/TAZ target. Notably, loss of EXT1 did not affect SLC35B2 levels (Supplementary Fig. 5b).

sgRNAs targeting EXT1 were depleted in Vemurafenib-treated SKMEL28-YAP^5SA^ cells (Supplementary Table 3) without reaching statistical significance (*p* = 0.06776; adj *p* = 0.554513; Supplementary Table 3) in the CRISPR/Cas9 screen, indicating potential functional relevance for MAPKi resistance. Indeed, similar to SLC35B2 knockout, loss of EXT1 reduced HS expression, albeit to a lesser extent, and markedly suppressed YAP^5SA^ cell proliferation in combination with Vemurafenib (Fig. 5h, i). Thus, the reliance of active YAP1 on high HS expression levels to achieve an efficient drug resistance phenotype can be utilized as a cancer vulnerability.

### Heparin antagonizes HS and sensitizes YAP^5SA^ melanoma cells to Vemurafenib

Heparin is structurally similar to HS, which allows its use for antagonizing HS by competition [44]. We therefore used heparin to test whether competitive HS inhibition re-sensitized resistant YAP^5SA^ melanoma cells to Vemurafenib. We treated cells with Vemurafenib only, unfractionated (UFH) or low molecular weight heparin (LMWH) only, or their combination, and observed that whereas each treatment alone had little effect on the proliferation of SKMEL28-YAP^5SA^ cells, the combination of Vemurafenib and UFH or LMWH more efficiently reduced cell proliferation (Fig. 5j).

### Loss of SLC35B2 and HS expression reduced receptor tyrosine kinase activity

**Fig. 6:**
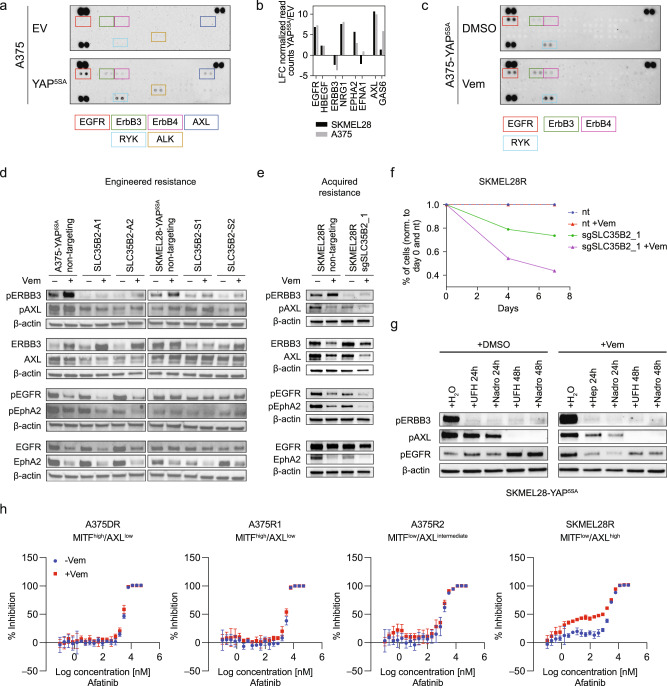
YAP1 broadly activates receptor tyrosine kinases in an SLC35B2-dependent manner. **a** Phospho-RTK array of A375-EV and A375-YAP^5SA^ cells. **b** Gene expression of selected RTKs and respective ligands in YAP^5SA^ cells and EV controls as assessed by RNA-seq (LFC, log_2_ fold change; RNA was isolated from two biological replicates per cell line). **c** Phospho-RTK array of A375-YAP^5SA^ cells treated for 24 h with 5 µM Vemurafenib or DMSO. **d**, **e** Immunoblotting of indicated antibodies using protein lysates from YAP^5SA^ (**d**) or non-engineered SKMEL28R (**e**) melanoma cells with SLC35B2 knockout and controls treated with Vemurafenib. **f** Growth competition assays of SKMEL28R cells with knockout of SLC35B2 compared to non-targeting controls with and without Vemurafenib treatment (10 µM). **g** Immunoblotting of indicated antibodies using protein lysates from SKMEL28-YAP^5SA^ cells treated with H_2_O, unfractionated heparin (UFH), and the low molecular weight heparin Nadroparin (Nadro; this experiment was performed once). **h** Cell viability assays to determine the effect of single treatment with the pan-ERBB inhibitor Afatinib and combination treatment with Vemurafenib in melanoma cells with acquired MAPKi resistance and different MITF/AXL expression levels (mean ± SD of three replicates is presented). Experiments in **a**, **c**–**f**, and **h** were performed at least two times independently, one representative experiment is shown.

We sought to identify the underlying mechanism by which loss of SLC35B2 and reduced HS levels re-sensitize YAP^5SA^ melanoma cells to Vemurafenib. As YAP1 stimulated the expression of RTKs and their ligands, we first assessed RTK activity in YAP^5SA^ melanoma cells compared to controls using phospho-RTK arrays. This revealed multiple RTKs activated in both YAP^5SA^ cell lines, including EGFR, ERBB3, AXL, and ALK, as well as additional cell line-specific RTKs (Fig. 6a and Supplementary Fig. 6). Interestingly, some of these RTKs and/or their ligands were strongly upregulated in YAP^5SA^ cell lines (Fig. 6b), suggesting YAP1-driven autocrine RTK activation loops. Whereas phospho-EGFR and phospho-AXL were either decreased or not affected by Vemurafenib treatment, pERBB3 and—to a lesser extent—pERBB4 were induced upon Vemurafenib treatment (Fig. 6c).

SLC35B2 knockout limited activation of RTKs that were activated by YAP1 including ERBB3 in both YAP^5SA^ cell lines and AXL in A375-YAP^5SA^ cells (Fig. 6d), indicating that their activity or activation depends on SLC35B2. As AXL and ERBB3 have been shown individually to contribute to Vemurafenib resistance [22, 23, 45–47], their reduced activity or activation in the absence of SLC35B2 can explain the re-sensitization effect of SLC35B2 knockout of YAP^5SA^ cells to Vemurafenib. Importantly, reduced activity of ERBB3, AXL, EPHA2, and EGFR was also observed after knockout of SLC35B2 in non-engineered Vemurafenib-resistant SKMEL28R melanoma cells with MITF^low^/AXL^high^ phenotype and high intrinsic YAP1/TAZ activity (Fig. 6e and Supplementary Fig. 7), indicating that the effect of SLC35B2 on RTK signaling was not specific for engineered YAP1 activation. The altered RTK activity pattern upon knockout of SLC35B2 was associated with re-sensitization toward Vemurafenib in growth competition assays, similar to the YAP^5SA^ cell lines (Fig. 6f).

Interestingly, treatment of SKMEL28-YAP^5SA^ cells with high doses of UFH or the LMWH nadroparin efficiently suppressed ERBB3 and AXL activity, comparably to loss of SLC35B2 (Fig. 6g), further supporting HS dependency of ERBB3 and AXL signaling in melanoma cells. Yet, EGFR was activated by UFH and LMWH, most likely due to direct EGFR activation by heparin, as described for other RTKs [48], which was overcome by Vemurafenib treatment. However, the HS dependency of EGFR activity has been demonstrated before [49].

The most prominent decrease in protein levels or RTK activity across cell lines was observed for ERBB3, followed by EGFR (also known as ERBB1) and AXL (Fig. 6d, e). To test whether this finding might be of translational relevance and ERBB inhibition might sensitize MAPKi-resistant melanoma cells to Vemurafenib, we treated melanoma cell lines with acquired MAPKi resistance and different MITF/AXL phenotypes with a combination of the pan-ERBB inhibitor Afatinib (Fig. 6h) and Vemurafenib, and observed a re-sensitization effect for the combination treatment in MITF^low^/AXL^high^ resistant melanoma cells with high RTK activity. In contrast, re-sensitization through combination treatment was not detected in resistant MITF^high^/AXL^low^ or MITF^low^/AXL^intermediate^ resistant melanoma cells without or with only modestly increased RTK activity. This supports the dependency of MAPKi resistance on active ERBB signaling in MITF^low^/AXL^high^ melanoma cells with high YAP1/TAZ activity.

**Fig. 7:**
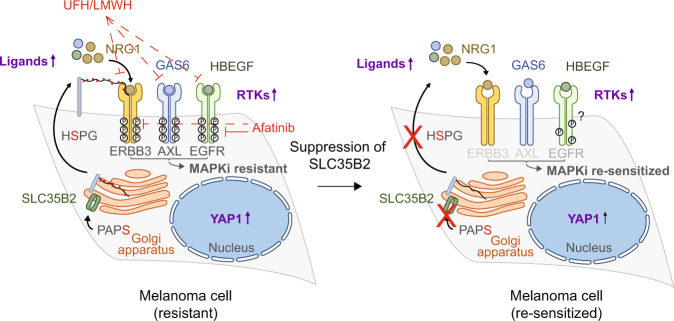
SLC35B2 is a vulnerability in YAP1-driven MAPKi-resistant, BRAF mutant melanoma. Graph illustrating the mechanism by which suppression of SLC35B2 re-sensitizes YAP1-driven melanoma cells to MAPK pathway inhibitors. YAP1-driven RTK activation through upregulation of RTKs and/or ligands mediates Vemurafenib resistance, the latter relying on heparan sulfate proteoglycans (HSPGs) for efficient RTK activation. In this context, heparan sulfation becomes critical for sufficient HSPG expression and loss of SLC35B2 abrogates HSPG synthesis by decreasing the transport of PAPS from cytosol into the Golgi apparatus. As a consequence, activity of multiple RTKs including ERBB3, AXL, and in part also EGFR is diminished, which is associated with re-sensitization toward Vemurafenib. Unfractionated heparin (UFH) and low molecular weight heparin (LMWH) can interfere with the interaction of HS, RTKs, and their ligands and thereby inhibit ERBB3, AXL, and (in the presence of Vemurafenib) EGFR activity, which re-sensitizes resistant melanoma cells toward MAPKis. The pan-ERBB inhibitor Afatinib inhibits the ERBB family of RTKs and shows MAPKi re-sensitization in MITF^low^/AXL^high^ melanoma cells that are characterized by high YAP1/TAZ activity.

In summary, we identify SLC35B2 as a novel vulnerability of YAP1-driven MAPKi resistance in melanoma through limiting heparan sulfation and thereby compensatory RTK activation (Fig. 7).

## Discussion

Using a genome-wide screen in melanoma cells with defined levels of YAP1 activity, we identified vulnerabilities associated with YAP1/TAZ activity in general and specifically in the context of MAPKi treatment. We highlight SLC35B2 whose strong overexpression in primary tumors and metastases compared to normal tissue makes it an attractive anti-cancer target. Across cancers, high SLC35B2 expression is associated with shorter PFI. In melanoma, however, this association is only significant for BRAF^V600E^ mutant tumors, suggesting a role of SLC35B2 particularly in the context of MAPKi treatment which represents a standard treatment in advanced BRAF^V600^ mutant, but not BRAF wildtype melanoma.

SLC35B2 is a sulfate transporter in the Golgi membrane and critical for sulfation of secreted or transmembrane proteins as well as proteoglycans, including HS [36]. HS was of particular interest, as it contributes to RTK activation by forming a complex with RTK ligands and their receptors [50]. RTKs mediate drug resistance by compensatory pathway activation upon treatment with targeted drugs [51, 52]. In YAP1-activated melanoma cells, SLC35B2 knockout abrogated HS surface expression and limited activity of YAP1-stimulated RTKs including ERBB3, EGFR, and AXL. The SLC35B2 knockout-dependent loss of HS expression was associated with re-sensitization of YAP1-activated melanoma cells toward MAPKis in vitro and in vivo. Thus, targeting heparan sulfation via SLC35B2 represents a conceptionally novel approach for suppressing RTK activity and overcoming RTK-mediated drug resistance.

Interestingly, YAP1/TAZ activity was associated with slight downregulation of SLC35B2, even though still highly overexpressed compared to normal tissue, which might induce a critical “bottleneck” when cells rely particularly on HS for increased RTK activity. The microRNA miR-22 can act as a tumor suppressor, is downregulated in many cancers including melanoma, and has been shown to suppress mRNA levels of SLC35B2 [53]. Thus, SLC35B2 overexpression might be a consequence of miR-22 suppression.

ERBB3, EGFR, and AXL can be activated by YAP1/TAZ and are well known to contribute to MAPKi resistance, which most likely explains the re-sensitization effect by SLC35B2 knockout toward MAPKis. Notably, pharmacological BRAF inhibition further stimulated ERBB3 signaling in YAP1-activated melanoma cells in line with a previous report [46], highlighting ERBB3 as a particularly relevant target in MAPKi-resistant melanoma cells with high YAP1 activity. While co-targeting of ERBB receptors has been described to enhance the efficacy of BRAF inhibition in melanoma [46], our data suggest that MITF^low^/AXL^high^ melanomas with high RTK activity might preferentially benefit from such a combination treatment in contrast to MAPKi-resistant melanoma without or with only limited RTK activity, which provides a molecular rationale for patient stratification.

To ensure high HS levels, YAP1/TAZ stimulate HS expression to a certain level via overexpression of EXT1 which has been previously considered an anti-cancer target [54]. However, we observed that HS expression was more efficiently abrogated upon SLC35B2 knockout compared to EXT1 knockout in melanoma cells, along with a significantly more pronounced antiproliferative effect of the respective knockout cells in presence of Vemurafenib, suggesting a higher therapeutic potential of SLC35B2 compared to EXT1. Notably, deleterious mutations in EXT1 cause the rare disorder Hereditary Multiple Exostoses, which is characterized by the development of benign bone tumors during childhood, a part of which spontaneously regress after adolescence [55]. The observation that most patients with this disorder are otherwise healthy provides evidence for the suitability of targeting HS for cancer therapy. However, SLC35B3—a paralog of SLC35B2—is expressed in the colon and might compensate for the loss of SLC35B2 activity in colorectal cancer [56].

SLC35B2 has not been evaluated as a target for anti-cancer therapies. Yet, its overexpression in tumors as well as its non-essentiality in normal cells define a therapeutic window. Currently, inhibitors for SLC35B2 are lacking. However, the availability of inhibitors for other proteins of the SLC family of transporters suggests similar “druggability” for SLC35B2 [57].

An impact of HS levels on the activity of multiple RTKs including ERBB, FGFR, and PDGFR family members as well as AXL and MET in tumor types apart from melanoma has been described [51, 58]. It is thus tempting to speculate that an RTK spectrum beyond ERBB3, EGFR, and AXL might be affected by SLC35B2 levels, depending on individual tumor type and treatment context.

While SLC35B2 knockout sensitized YAP1-activated melanoma cells toward Vemurafenib treatment, it did not completely stop melanoma cell proliferation, comparable to the strong, but limited effect of Vemurafenib single treatment on BRAF^V600^ mutant melanoma cells without YAP1 activation. Thus, SLC35B2 inhibition might require the addition of simultaneous BRAF and MEK inhibition to achieve the maximum growth inhibition.

We demonstrate that YAP1/TAZ activity correlates positively with resistance to MAPKis across a large panel of melanoma cell lines in vitro, irrespective of additional genetic aberrations, and with the MITF^low^/AXL^high^ phenotype across melanoma cells lines and patient tumors, a subset of which acquires increased YAP1/TAZ activity along with MAPKi resistance. YAP1 induces transcription of multiple RTKs and RTK signaling by overexpression of RTK ligands such as GAS6, HB-EGF, and NRG1. Interestingly, YAP1-driven RTK overexpression reflects a melanoma stem-cell-like phenotype and YAP1-mediated MAPKi resistance relies on molecular programs that characterize stem cell-like cellular states [59].

In summary, we describe high YAP1/TAZ activity as a molecular driver of MAPKi-resistant MITF^low^/AXL^high^ melanomas and identify SLC35B2 as a single target for overcoming broad compensatory pathway activation through limiting RTK activity. The MITF^low^/AXL^high^ phenotype might serve as a biomarker for identifying RTK-driven resistant melanomas that are particularly likely to respond to a co-treatment approach targeting the MAPK pathway and heparan sulfation.

## Materials and methods

### Cell lines

A375 (in-house stocks [Prof. Reuven Agami’s lab], originally from ATCC [Manassas, VA, United States; CRL-1619], SKMEL28 (in-house stocks [Prof. Reuven Agami’s lab]) and HEK 293T (in-house stocks [Prof. Reuven Agami’s lab], originally from ATCC [CRL-3216]) cells were cultured in DMEM medium (Gibco, Waltham, MA, United States), supplemented with 1% Penicillin/Streptomycin (Gibco) and 10% fetal calf serum (FCS; Hyclone, Logan, UT, United States). A375DR, A375R1, A375R2, and SKMEL28R cells were a gift from Prof. Daniel Peeper (The Netherlands Cancer Institute, Amsterdam, The Netherlands), generated as described previously [23] and were cultured in DMEM medium, supplemented with 1% Penicillin/Streptomycin, 10% FCS and 1 μM Vemurafenib (PLX4032, Selleckchem, Houston, TX, United States). Cell lines were regularly tested negative for Mycoplasma.

### Mice models

Mice were bred and maintained in accordance with all applicable laws and regulations subsequent to approval by the Netherlands Cancer Institute’s animal care and ethical committee. In all, 1 × 10^6^ tumor cells were injected subcutaneously in 6–8-week-old NOD.Cg-*Prkdc*^*scid*^*Il2rg*^*tm1Wjl*^/SzJ (NSG) male mice. Tumors were measured three times per week by caliper. When tumors reached a volume of 100 mm^3^, Vemurafenib (50 mg kg^–1^ per day by oral gavage) or respective vehicle treatment was started. When tumors reached a volume of 1500 mm^3^ or after a maximum of 6 months, mice were sacrificed and tumors were collected. Statistical analysis of tumor growth was performed by application of a mixed-effects model fit, with mouse effect random and treatment effect fixed, using the R package “qvcalc” (https://CRAN.R-project.org/package=qvcalc).

### YAP1 activity reporter assay

For assessment of YAP1 activity, 1 × 10^5^ cells were seeded in 6-well plates at least in triplicates, co-transfected on the following day with 200 ng of a YAP1 luciferase reporter described previously [60] and 20 ng Renilla as transfection control using 1 µg Polyethyleneimine “MAX” (Polysciences, Warrington, PA, United States) per µg DNA. Luciferase activity was measured 24 h post-transfection using the Dual-Luciferase Reporter assay kit (Promega, Madison, WI, United States) according to the manufacturer’s protocol and a Centro XS3 LB960 machine (Berthold Technologies, Bad Wildbad, Germany) or an Infinite M200 PRO (Tecan, Männedorf, Switzerland). Luciferase activity was normalized to Renilla. Each experiment was performed at least twice on different days.

### Drug assays

Melanoma cells were seeded at a density of 5 × 10^3^ cells in 100 µl medium per well of a 96-well plate at least in triplicates (Greiner, Kremsmünster, Austria). On the following day, cells were treated with 9-point dilutions of Vemurafenib (100 µM to 1 nM), Cobimetinib (1 µM to 10 pM; Selleckchem) or DMSO (Sigma-Aldrich, St. Louis, MO, United States) for 4 days. Finally, 10 µl Resazurin (Serva, Heidelberg, Germany) was added to each well. After 2 h incubation, fluorescence intensity was measured using a Tecan reader. Alternatively, the ATP^lite^ luminescence assay (Perkin Elmer, Waltham, MA, United States) was used as described before [61]. For combination treatment of Afatinib (MedChemExpress, Monmouth Junction, NJ, United States) with Vemurafenib, a fixed Vemurafenib dose was used and 20-point dilutions of Afatinib (50 µM to 95 pM) were performed as indicated in the respective figure legends. Dose-response curves were calculated using Prism (GraphPad, San Diego, CA, United States; Versions 8/9). Drug assays were performed at least twice on different days, representative results are presented.

For combination treatments of UFH or LMWH with Vemurafenib, 1 × 10^4^ cells were seeded per well of a 6-well plate in duplicates and treated the next day with indicated concentrations of Heparin, Nadroparin (all Sigma-Aldrich) and/or Vemurafenib as indicated. Medium including drugs was changed every three to four days and all cells were split at the same ratio when DMSO-treated control cells reached subconfluence. After 15 days of treatment, cells were fixed and crystal violet staining was performed as detailed below. This experiment was performed twice on different days, representative results are presented.

### Immunoblotting

For protein isolation, cells were lysed in an appropriate volume of RIPA buffer supplemented with protease and phosphatase inhibitors (all Thermo Fisher, Waltham, MA, United States). Protein concentration was measured using Pierce BCA protein assay kit (Thermo Fisher) and adjusted to 10–50 µg per lane, using 4× Laemmli Sample Buffer with the addition of 10% 2-mercaptoethanol (all Bio-Rad, Hercules, CA, United States). Samples were run on 4–15% Mini-Protean TGX pre-cast protein (Bio-Rad) or SDS-PAGE gels and transferred to polyvinylidene fluoride (PVDF, Bio-Rad) or 22 mm pore size nitrocellulose membranes (Santa Cruz, Santa Cruz, CA, United States). Membranes were blocked for 1 h at room temperature (RT) using 4% milk in TBS-T (20 mM Tris-HCl pH 7.5, 150 mM NaCl, 0.1% Tween20), residual blocking buffer was removed and membranes were incubated in the primary antibody at 4 °C overnight. Membranes were incubated with HRP-conjugated secondary antibody or LI-COR secondary antibodies at RT for 1 h. For imaging, blots stained with HRP-conjugated secondary antibodies were incubated in Clarity Max Western ECL Substrate or Clarity Western ECL Substrate and developed using the ChemiDoc MP Imaging System (all Bio-Rad). Blot stained with LI-COR secondary antibodies were visualized by use of an Odyssey infrared scanning device (LI-COR, Lincoln, NE, United States). The following antibodies were used: YAP1 (#ab52771; abcam, Cambridge, United Kingdom; 1:1000 or #sc-101199; Santa Cruz; 1:1000), pMEK (#9154; CST, Danvers, MA, United States; 1:1000), MEK (#4694; CST; 1:1000), pEGFR (#44-788G; Thermo Fisher; 1:1000), EGFR (#2239; CST; 1:1000), pERBB3 (#4791; CST; 1:1000), ERBB3 (#12708; CST; 1:1000), pEPHA2 (#3970; CST; 1:1000), EPHA2 (#6997; CST; 1:1000), pAXL (#5724; CST; 1:1000), AXL (#8661; CST; 1:1000), HSP90 (#610418; BD Biosciences, Franklin Lakes, NJ, United States; 1:5000), GAPDH (#sc-32233; Santa Cruz; 1:2000), β-ACTIN (#A3854, Sigma-Aldrich; 1:1000), SOX10 (#ab155279; abcam; 1:000), MITF (#97800; CST; 1:1000), TAZ (#8418; CST; 1:1000), EXT1 (#sc-515144; Santa Cruz; 1:1000). Secondary antibodies: goat anti-rabbit IgG H&L (HRP; #ab6721; abcam; 1:10,000), rabbit anti-mouse IgG H&L (HRP; #ab6728; abcam; 1:10,000), 680RD donkey anti-mouse secondary antibody (#926-68072; LI-COR), 680RD donkey anti-rabbit secondary antibody (#926-68073; LI-COR), 800CW goat anti-mouse secondary antibody (#926-32350; LI-COR), 800CW goat anti-rabbit secondary antibody (#926-32211; LI-COR). Immunoblots were performed at least twice with independent protein lysates unless stated otherwise.

### RNA-seq analysis and YAP1/TAZ score calculation

Total RNA was isolated from A375-YAP^5SA^, SKMEL28-YAP^5SA^, and respective EV control cells with two replicates per cell line using Trizol (Thermo Fisher). Strand-specific libraries were generated per sample using the TruSeq Stranded mRNA sample preparation kit (#RS-122-2101/2; Illumina, San Diego, CA, United States) according to the manufacturer’s instructions. The generated cDNA fragments were 3’-end adenylated and ligated to Illumina paired-end sequencing adapters and subsequently amplified by 12 cycles of PCR. The libraries were sequenced with 65 base single reads on a HiSeq2500 using V4 chemistry (Illumina).

RNA-seq data, as FASTQ files, were aligned to the human hg19 genome using TopHat [62]. SAMtools was used for file format conversions [63]. HTSeq was used to count reads at exons of protein-coding genes [64]. Library size normalization of read counts was done using DESeq [65].

GO term enrichment analysis was performed with Toppgene (accessed via https://toppgene.cchmc.org on December 14, 2021) and genes upregulated in both YAP^5SA^ cell lines (adj *p* < 0.01, normalized read count >200 in both YAP^5SA^ cell lines) were used as input. GSEA was performed using pre-ranked GSEA with the metric −log_10_(*p* value) * sign(LFQ differences) using GSEA software [66] and previously defined gene sets for MITF targets [67], proliferative and invasive genes [68], and NF-κB targets [69, 70].

For cell lines and patient tumors, a YAP1/TAZ score was calculated as described [28]. In short, a summary z-score was calculated by adding individual *z*-scores of the 22 target genes from the YAP1/TAZ target gene signature established by Wang et al.

### Correlation of gene expression and drug sensitivity

For RNA-seq analysis of melanoma cell lines, RSEM normalized gene data was obtained from the CCLE (accessed via https://sites.broadinstitute.org/ccle/datasets) [29]. Melanoma cell lines were filtered for BRAF^V600E^ mutations using genotyping data from CCLE. Drug sensitivity data were obtained from CCLE.

For gene expression analysis of TCGA data, normalized gene data from the SKCM dataset were obtained from https://portal.gdc.cancer.gov/. RNA-seq data from baseline and resistant melanoma tumors [30] were accessed via GEO: GSE65186 and analyzed as detailed above.

For the visualization, the pheatmap R package was used with a *z*-score calculated among all included genes and sensitivity markers. A Pearson correlation coefficient was calculated using the YAP1/TAZ score as an index.

### Lentivirus production and infection

For lentivirus production, HEK 293T cells were seeded at a density of 4 × 10^6^ cells per 10 cm dish one day prior to transfection. For transfection, 10 μg of the plasmid of interest, 5 μg pMDL RRE, 3.5 μg pVSV-G, and 2.5 μg pRSV-REV plasmids were mixed in 500 μl serum-free DMEM. Next, 500 μl serum-free DMEM containing 63 μl of 1 mg ml^–1^ Polyethylenimine (PEI; Polysciences) was added. The DNA/PEI mix was vortexed and incubated for 15 min at RT, and subsequently added to the HEK 293T cells. The next day, the medium was replaced. The lentivirus-containing supernatant was collected 48 and 72 h post transfection and snap-frozen in liquid nitrogen. Target cells were transduced by supplementation of the lentiviral supernatant with 8 µg ml^–1^ Polybrene (Sigma-Aldrich). Infected cells were selected by addition of 2 µg ml^–1^ Puromycin (Jena Bioscience, Jena, Germany) 48 h after infection until no living cells were left in a control plate that had not been transduced.

### sgRNA library cloning

The human lentiCRISPR-v2 knockout pooled library (Brunello) was a gift from David Root and John Doench (addgene, Watertown, MA, USA; plasmid #73178) [32]. Endura electrocompetent cells (Lucigen, Middleton, WI, United States) were transformed with Brunello library plasmids as described [71]. Plasmid DNA was prepared from bacterial cultures using PureLink HiPure Plasmid Maxiprep-Kit (Qiagen, Hilden, Germany). Lentivirus production, infection, and selection of infected cells were performed as described above.

### Genome-wide CRISPR/Cas9 screen

Per screen condition, three replicates of 6 × 10^7^ SKMEL28-YAP^5SA^ or SKMEL28-EV cells were seeded and transduced on the following day with lentiviral pools of the pLentiCRISPRv2 Brunello library, respectively (multiplicity of infection 0.3). After selection with Puromycin, at least half of the transduced cells were harvested at day 0 for genomic DNA isolation, while the remaining SKMEL28-YAP^5SA^ cells were cultured for 14 days in the absence or 21 days in the presence of 5 µM Vemurafenib (PLX4032; #S1267; Selleckchem, Houston, TX, United States) and the remaining SKMEL28-EV cells were propagated without Vemurafenib for 21 days to ensure the same number of cell divisions in all conditions. Afterward, cells were harvested, genomic DNA was isolated using a lysis buffer (10% v/v 1 M Tris-HCl pH 8, 1% v/v 0.5 M EDTA, 2% v/v 1 M NaCl, 20% v/v SDS (10% w/v), 67% v/v water), and DNA was purified after digestion of samples with RNAse A (Purelink, Thermo Fisher) and Proteinase K (Roche, Basel, Switzerland) using phenol (Acros, Waltham, MA, United States) and chloroform (Serva). sgRNAs were PCR-amplified using 15 cycles per PCR and submitted for next-generation sequencing as described [72]. Sequencing was done using single reads of 65 bp on the HiSeq2500 platform (Illumina).

### CRISPR/Cas9 screen analysis

Read counts per sgRNA were obtained from raw read count files using the caRpools library in R [73]. Read counts per sgRNA were then calculated using R. To assess relative changes between starting and final cell population, MAGeCK software was employed using nt sgRNAs for normalization [74].

### Individual gene knock-outs

From the genome-wide CRISPR/Cas9 screen 12 genes were selected for which all four sgRNAs were significantly depleted only in Vemurafenib-treated SKMEL28-YAP^5SA^ cells, but not in other conditions. The individual sgRNAs were cloned into a lentiCRISPR-v2 (addgene, plasmid #52961) vector as described [75]. Lentivirus production, transduction, and selection were performed as described above.

### SLC35B2 knockout clones

For the generation of SLC35B2 knockout clones, single cells transduced with sgRNAs targeting SLC35B2 and selected with Puromycin were picked and expanded. Efficient genome editing was confirmed using Sanger sequencing of PCR amplicons (primers see Supplementary Table 4) of the expected cutting site. Quantitative analysis of indels around the cutting site was performed using TIDE software (http://tide.nki.nl) [76].

### SLC35B2 overexpressing cells

For overexpression of an SLC35B2 version resistant to SLC35B2-targeting sgRNAs, A375-YAP^5SA^ SLC35B2 knockout clones were transduced with a codon-optimized (co) SLC35B2 ORF (gift from RESOLUTE Consortium & Giulio Superti-Furga [addgene, plasmid #132251]) in a plentiCDH-Hygro backbone. Transduced cells were selected with 200 µg ml^–1^ Hygromycin B (Thermo Fisher). Expression of functional SLC35B2 was confirmed by the rescue of HS expression in SLC35B2 knockout cells.

### eGFP competition assays

SKMEL28-YAP^5SA^ and A375-YAP^5SA^ cells were infected with an eGFP-encoding lentiviral vector and selected with Puromycin. eGFP-expressing cells were mixed in a 1:3 to 1:4 ratio with SKMEL28-YAP^5SA^ and A375-YAP^5SA^ cells containing individual sgRNAs, respectively, and were cultivated in the presence or absence of 5 µM Vemurafenib. Vemurafenib was added every 2–3 days. The percentage of eGFP-expressing cells was assessed by flow cytometry at the beginning of the experiment (day 0) and at day 11 and normalized to the nt control. For every condition, at least 10,000 events were recorded using an Attune NxT flow cytometer (Thermo Fisher) or a BD Aria II (BD) and data were analyzed using FlowJo and FACSDiva software (both BD). Competition assays were performed in duplicates.

### Incucyte proliferation assay

A total of 500 cells were seeded at least in triplicates in 80 µl of medium per well of 96-well plates (Greiner). After 4 h, 20 µl DMEM supplemented with 25 µM Vemurafenib or DMSO were added to the wells. Cells were imaged every 4 h using the Incucyte ZOOM (Essen Bioscience, Ann Arbor, MI, United States). Phase-contrast images were analyzed to detect cell proliferation based on cell confluence. Outliers due to batch effects were removed. Proliferation assays were performed at least twice. Representative results are presented.

### Crystal violet staining

In all, 1 × 10^4^ SKMEL28-YAP^5SA^ and A375-YAP^5SA^ cells were seeded per well of a 6-well plate in duplicates and cultivated for 2 weeks. Cells were split when control cells were reaching subconfluence. Cells were treated with 5 µM Vemurafenib, 100 or 500 µg ml^–1^ heparin or the LMWH Nadroparin, a combination of Vemurafenib and heparin/Nadroparin, or left untreated as a control. The difference in cell growth was measured by crystal violet staining. Cells were washed with phosphate-buffered saline (PBS; Gibco), fixed with 4% formaldehyde in PBS for 10 min at RT, and washed with 1 ml dH_2_O and stained with 0.1% crystal violet in water for 30 min at RT. Plates were washed multiple times in water, dried, and scanned by a dual lens scan system (V750 PRO; Epson, Suwa, Japan). This experiment was performed twice.

### HS expression analysis

For assessment of HS surface expression, cells were stained with a primary antibody for 1 h at 4 °C (#370255-1; AmsBio, Abingdon, United Kingdom; 1:50), followed by two times washing in PBS supplemented with 5% FBS and staining with a goat anti-mouse antibody for 1 h at 4 °C in the dark (Alexa Fluor 488 AffiniPure Goat Anti-Mouse IgG + IgM [H+L]; #115-545-044, Jackson ImmunoResearch Laboratories Inc, West Grove, PA, United States; 1:50). After further washing steps, cells were resuspended in PBS supplemented with 5% FBS and 1 µg ml^–1^ DAPI (D1306; Invitrogen, Waltham, MA, United States) and recorded on an Attune NxT flow cytometer or a BD Aria II (BD). At least 10,000 events were recorded. As negative controls, samples were stained without the primary antibody. Experiments were performed at least twice, representative results are presented.

### Phospho-RTK arrays

The Proteome Profiler Human Phospho-RTK Array Kit (R&D Systems, Minneapolis, MN, United States) was used according to the manufacturer’s protocol and 250 µg protein lysate was used per sample. Membranes were recorded using a ChemiDoc MP Imaging System. Arrays were performed once per cell line and condition.

### qRT-PCR

Total RNA was obtained using the RNeasy plus mini kit (Qiagen). One microgram of RNA was reverse transcribed using the RevertAid First Strand cDNA Synthesis Kit (Thermo Fisher Scientific). qRT-PCR was performed with the Power SYBR™ Green PCR Master Mix (Applied Biosystems, Waltham, MA, United States) and analyzed using LightCycler 480 (Roche). Relative expression values for each gene of interest were obtained by normalizing to GAPDH or beta-Actin mRNA expression using the ΔΔCt method.

### Clinical outcome data analysis

Expression analysis of SLC35B2 across normal and tumor tissues and correlation with clinical outcome was performed using UCSC Xena browser (https://xenabrowser.net) [41]. Datasets were used as indicated in the respective figures or figure legends. For SLC35B2 expression across sample types (Fig. 4f), the “TCGA TARGET GTEx” study was used. TARGET cases (childhood cancers) were removed. RSEM normalized read counts are shown. For comparison across primary tumor types (Fig. 4g) as well as association with disease-specific survival and PFI (Fig. 4h, i), the same dataset was used and filtered for “TCGA” tumors. “Normal” as well as “control” samples were removed. For the association of SLC35B2 expression and melanoma outcome (Fig. 4j), the TCGA melanoma “SKCM” dataset was used and filtered for primary and metastatic tumors. PFI was shown separately for melanoma with and without BRAF^V600E^ mutation, respectively.

### Quantification and statistical analysis

Statistical analyses and graphical presentations were performed using Prism (versions 8/9, GraphPad). Statistical assays performed are specified in the figure legends.

## Supplementary information

Supplementary Information

Supplementary Table 1

Supplementary Table 2

Supplementary Table 3

Supplementary Table 4

## Data Availability

The data that support the findings of this study are available from the corresponding author upon reasonable request. Raw and processed RNA-seq data are deposited under GSE193775.
