# Supplementary material for: Suppression of heparan sulfation re-sensitizes YAP1-driven melanoma to MAPK pathway inhibitors
Source: Oncogene. 2022 Jul 7;41(32):3953–68. doi: 10.1038/s41388-022-02400-z (PMC9355870; doi:10.1038/s41388-022-02400-z)
Supplement: Supplementary file 1 — Supplementary Information [file 41388_2022_2400_MOESM1_ESM.pdf]

Supplementary Information:

Supplementary Figures:

Supplementary Figure 1: Genes correlated with MAPKi resistance are enriched in YAP^{5SA} melanoma cells.

Supplementary Figure 2: Established YAP1/TAZ target gene signatures are enriched in YAP^{5SA} melanoma cells.

Supplementary Figure 3: Validation of hits from CRISPR/Cas9 screen identifies EGFR and SLC35B2 as promising targets.

Supplementary Figure 4: Assessment of genome editing efficiency of SLC35B2 knock-out clones reveals loss of wildtype sequence.

Supplementary Figure 5: HS is highly expressed in SKMEL28-YAP^{5SA} and control cells.

Supplementary Figure 6: YAP1 broadly activates receptor tyrosine kinases in an SLC35B2-dependent manner.

Supplementary Figure 7: SLC35B2 knock-out decreases HS expression in SKMEL28R cells.

Supplementary Tables:

Supplementary Table 1 (separate Excel file): RNA-seq results from A375- and SKMEL28-YAP^{5SA} and -EV control cells.

Supplementary Table 2 (separate Excel file): YAP1 scores for melanoma cells lines and melanoma patient tumors.

Supplementary Table 3 (separate Excel file): CRISPR/Cas9 screen results.

Supplementary Table 4 (separate Excel file): Primers and oligonucleotides.

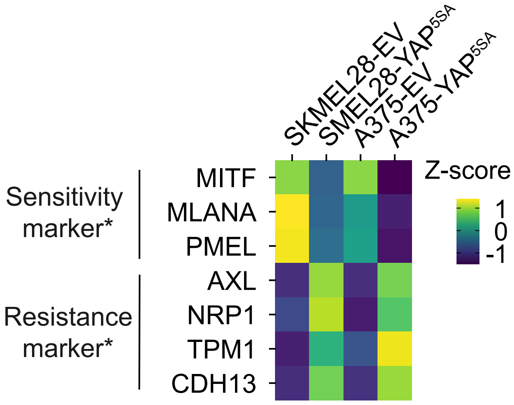

Supplementary Figure 1 | Genes correlated with MAPKi resistance are enriched in YAP^{5SA} melanoma cells. Genes are labeled as sensitivity or resistance marker genes (asterisks) according to their correlation with sensitivity or resistance to BRAF and MEK inhibitors in the CCLE collection of BRAF^{V600E} mutant melanoma cell lines *in vitro* as described in Konieczkowski *et al.* [1]. TYRP1 is not shown as it was barely expressed in EV or YAP^{5SA} cells. RNA was isolated from two biological replicates per cell line.

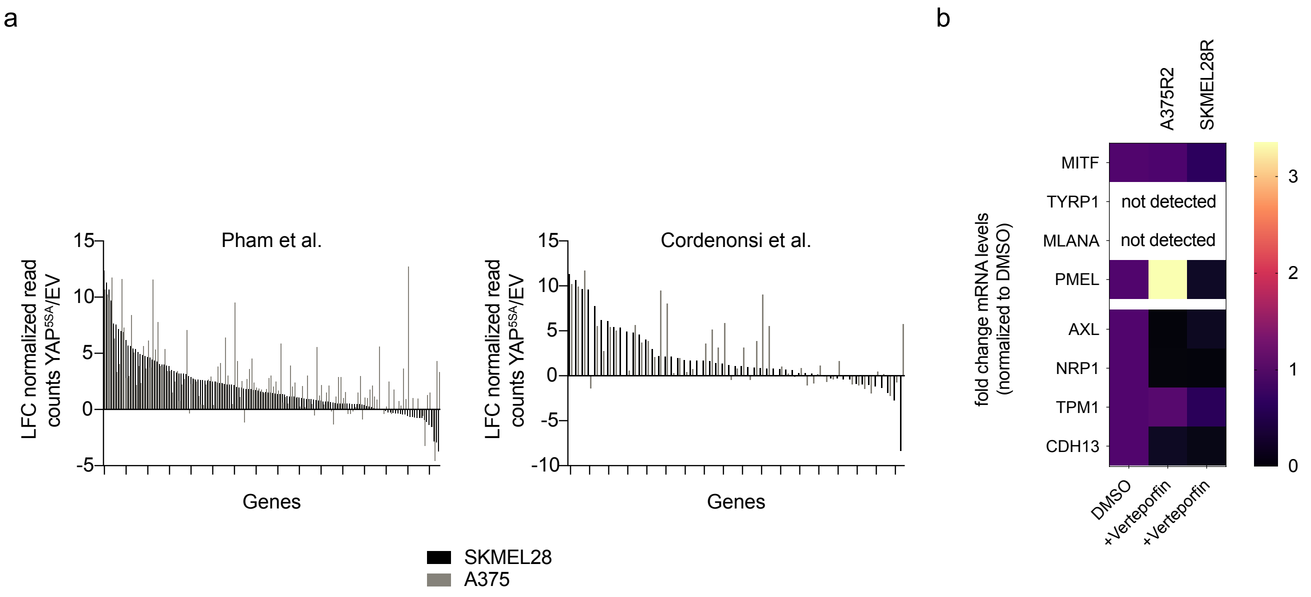

Supplementary Figure 2 | Established YAP1/TAZ target gene signatures are enriched in YAP^{5SA} melanoma cells. **a**, Expression of genes that are part of two additional YAP1 target gene signatures and were defined in Pham *et al.* [2] or Cordenonsi *et al.* [3]. $\log_2$ fold changes (LFC) of gene expression based on mRNA sequencing of YAP^{5SA} melanoma cells and controls are presented. RNA was isolated from two biological replicates. **b**, Fold changes in gene expression of sensitivity and resistance marker genes in MAPKi resistant melanoma cells with high intrinsic YAP1/TAZ activity after treatment with 10 µM Verteporfin for 24h as assessed by qPCR (mean of two replicates is shown).

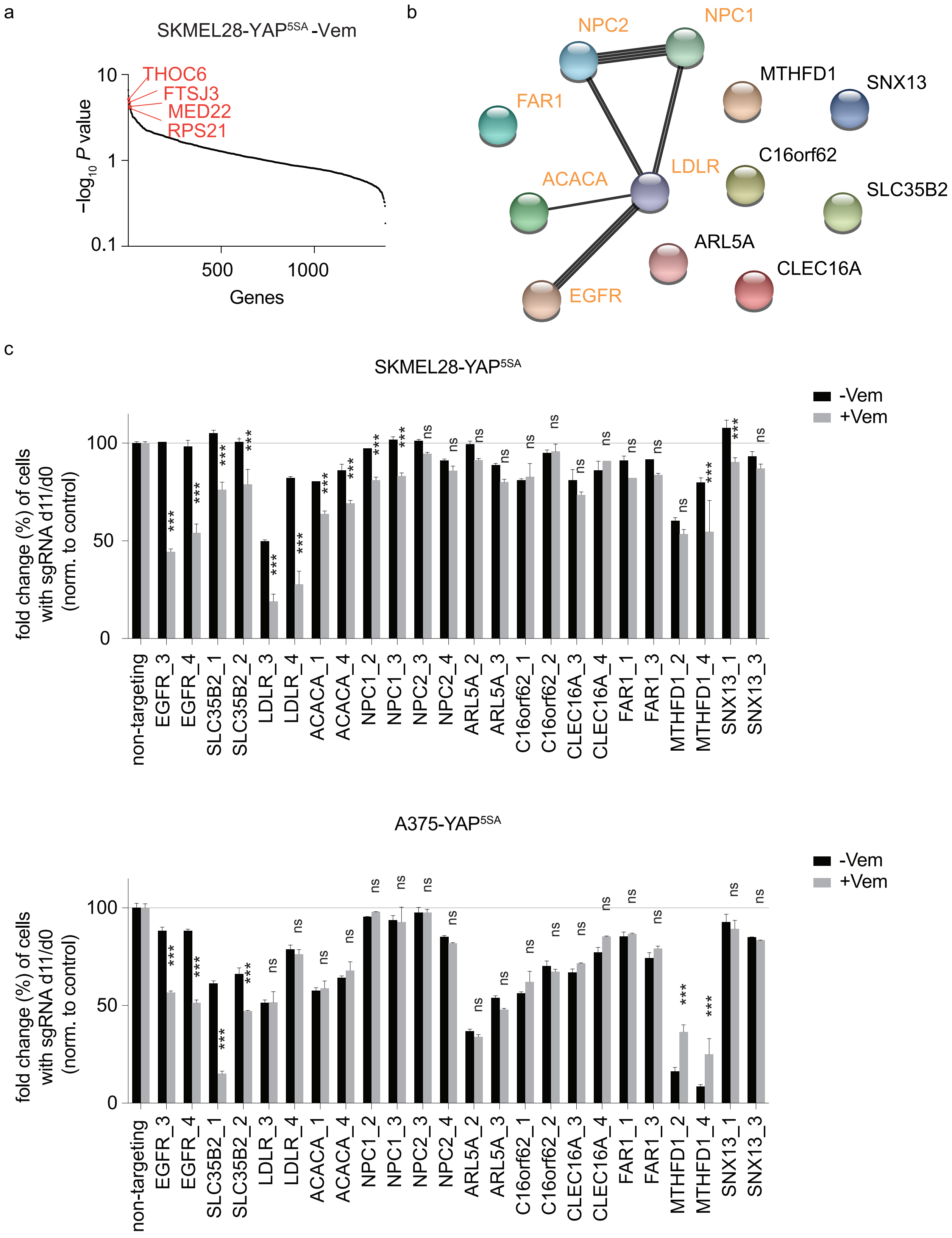

Supplementary Figure 3 | Validation of hits from CRISPR/Cas9 screen identifies EGFR and SLC35B2 as promising targets. a, Essential genes with respective sgRNAs being depleted in untreated SKMEL28-YAP[5SA], but not SKMEL28-EV cells.

b, Protein-protein interactions of hits from genome-wide CRISPR/Cas9 screen in SKMEL28-YAP^{5SA} cells identified as essential only in YAP1 activated cells, but not EV controls, and only in presence of Vemurafenib were visualized using STRING (accessed via https://string-db.org). Genes associated with lipid metabolism are labeled in orange. Number of lines between proteins corresponds to levels of evidence for direct protein-protein interactions. **c,** SKMEL28-YAP^{5SA} and A375-YAP^{5SA} cells were infected with an eGFP-encoding lentiviral vector and selected with Puromycin. eGFP expressing cells were mixed in a 1:3 to 1:4 ratio with SKMEL28-YAP^{5SA} and A375-YAP^{5SA} cells containing the individual sgRNAs, respectively, and were cultivated in the presence or absence of 5 μM Vemurafenib. The percentage of eGFP-expressing cells was assessed by flow cytometry at the beginning of the experiment (day 0) and at day 11 and the fold change of the eGFP ratio at day 11 compared to day 0 was calculated. These values were normalized to the non-targeting control (mean + SD from two replicates is presented. *p* values from unpaired, two-tailed *t*-tests comparing cells treated with 5 μM Vemurafenib compared to untreated cells. ***$p < 0.001$). This experiment was performed two times independently, one representative experiment is shown.

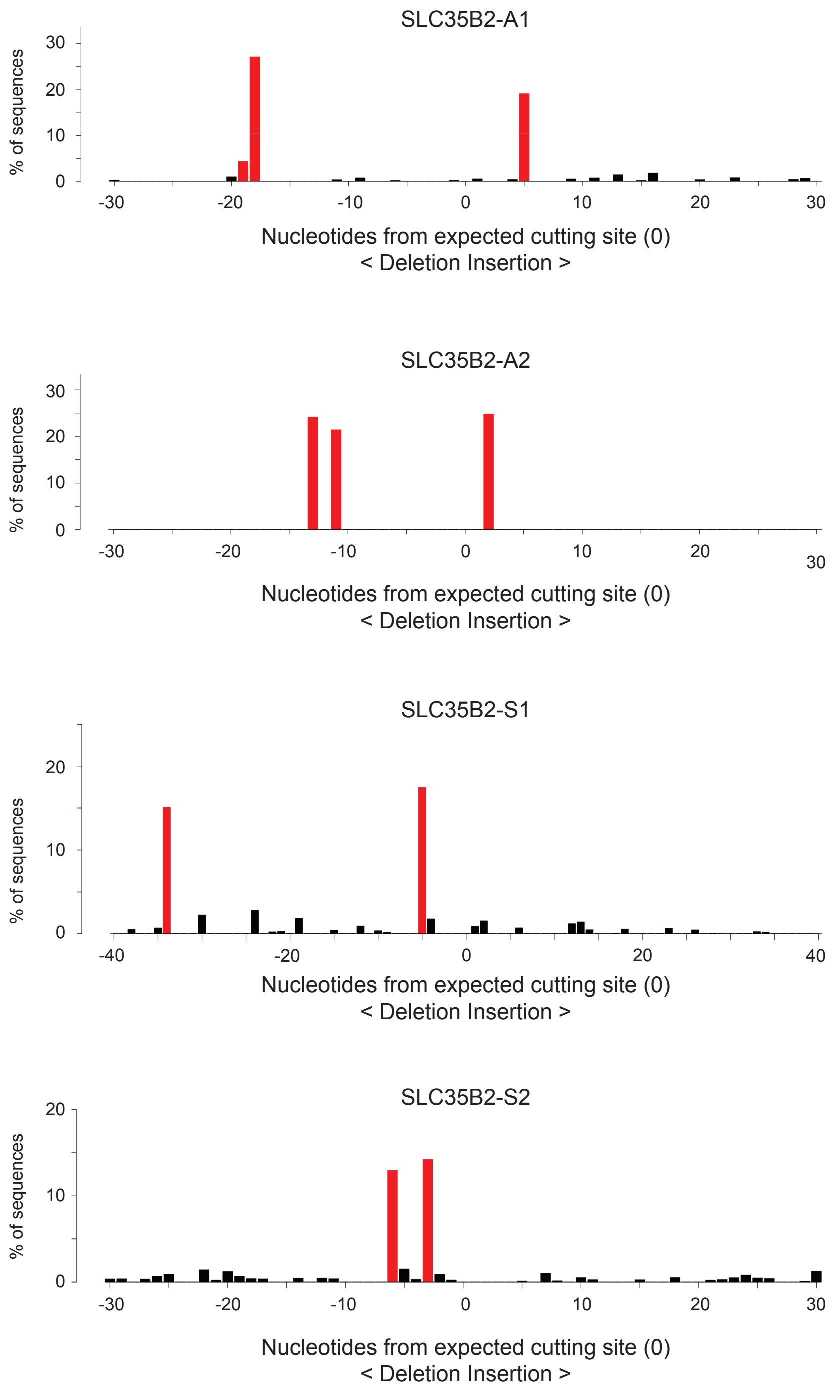

Supplementary Figure 4 | Assessment of genome editing efficiency of SLC35B2 knock-out clones reveals loss of wildtype sequence. PCR-amplified genomic sequences around the sgRNA cutting sites were sequenced by Sanger sequencing and the mutational load in SLC35B2 knock-out clones was calculated using TIDE (accessed via https://tide.nki.nl).

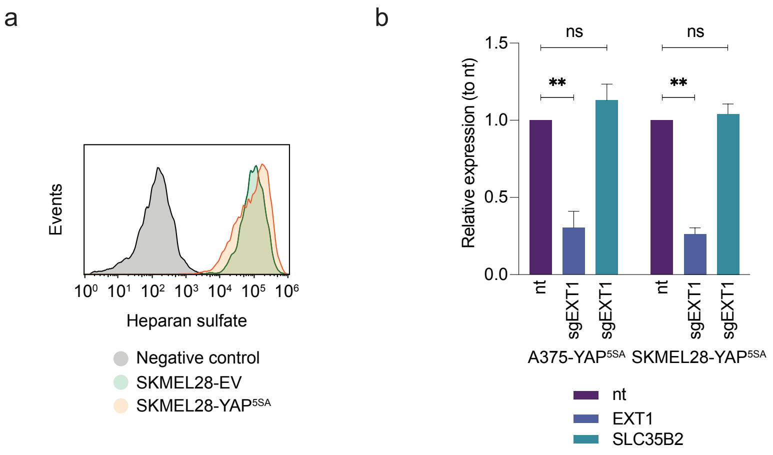

Supplementary Figure 5 | HS is highly expressed in SKMEL28-YAP^{5SA} and control cells. a, Flow cytometry-based assessment of HS cell surface expression in SKMEL28-YAP^{5SA} cells and empty vector (EV) controls (negative control, no primary antibody; this experiment was performed two times independently, one representative experiment is shown). **b,** mRNA expression levels of EXT1 and SLC35B2 after CRISPR/Cas9 mediated knock-out of EXT1, as quantified by qPCR and normalized to non-targeting (nt) controls (*p*resented are mean + SD from three replicates; *p* values from Welch's t-test; $^{**}p < 0.01$).

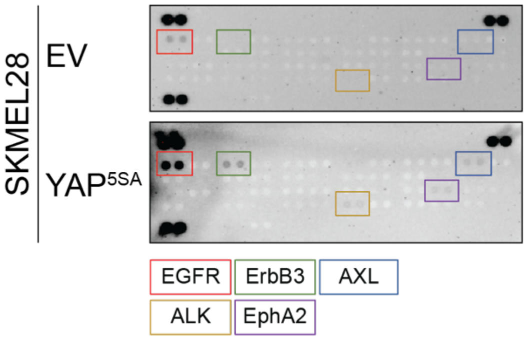

Supplementary Figure 6 | YAP1 broadly activates receptor tyrosine kinases in an SLC35B2-dependent manner. Phospho-RTK array of SKMEL28-EV and SKMEL28-YAP^{5SA} cells. This experiment was performed two times independently, one representative experiment is shown.

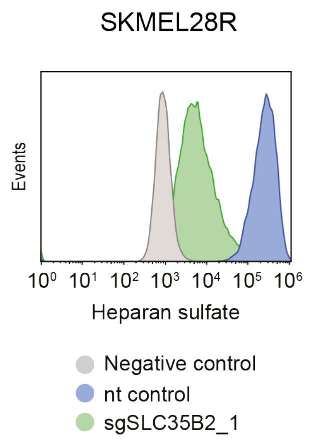

Supplementary Figure 7 | SLC35B2 knock-out decreases HS expression in SKMEL28R cells. Flow cytometry-based assessment of HS cell surface expression in SKMEL28R cells after CRISPR/Cas9 mediated knock-out of SLC35B2 compared to non-targeting (nt) control (negative control, no primary antibody). This experiment was performed two times independently, one representative experiment is shown.

References:

1 Konieczkowski DJ, Johannessen CM, Abudayyeh O, Kim JW, Cooper ZA, Piris A *et al*. A melanoma cell state distinction influences sensitivity to MAPK pathway inhibitors. *Cancer Discov* 2014; 4: 816-827.

2 Pham TH, Hagenbeek TJ, Lee HJ, Li J, Rose CM, Lin E *et al*. Machine-Learning and Chemicogenomics Approach Defines and Predicts Cross-Talk of Hippo and MAPK Pathways. *Cancer Discov* 2021; 11: 778-793.

3 Cordenonsi M, Zanconato F, Azzolin L, Forcato M, Rosato A, Frasson C *et al*. The Hippo transducer TAZ confers cancer stem cell-related traits on breast cancer cells. *Cell* 2011; 147: 759-772.
